# Chronic High Risk Prescription Opioid Use Among Persons With HIV

**DOI:** 10.3389/fsoc.2021.645992

**Published:** 2021-05-18

**Authors:** Ana Ventuneac, Gavriella Hecht, Emily Forcht, Bianca A. Duah, Shafaq Tarar, Blanche Langenbach, Jay Gates, Demetria Cain, H. Jonathon Rendina, Judith A. Aberg, David C. Perlman

**Affiliations:** ^1^ Icahn School of Medicine at Mount Sinai, Division of Infectious Diseases, NY, NY, United States; ^2^ Department of Psychology, Hunter College, City University of New York, NY, NY, United States; ^3^ Health Psychology and Clinical Science PhD Program, The Graduate Center, City University of New York, NY, NY, United States

**Keywords:** opioid prescription, morphine equivalent daily dose, chronic opioid therapy, HIV, viral suppression

## Abstract

Persons with HIV (PWH) are a population at risk for adverse sequelae of opioid use. Yet, few studies have examined correlates of chronic high risk opioid use and its impact on HIV outcomes. Trends in prescribing patterns and identification of factors that impact the use of opioid prescriptions among PWH are crucial to determine prevention and treatment interventions. This study examined electronic medical records (EMR) of patients receiving HIV care to characterize prescribing patterns and identify risk factors for chronic high risk prescription opioid use and the impact on HIV outcomes among PWH in primary care from July 1, 2016–December 31, 2017. EMR were analyzed from 8,882 patients who were predominantly male and ethnically and racially diverse with half being 50 years of age or older. The majority of the 8,744 prescriptions (98% oral and 2% transdermal preparations) given to 1,040 (12%) patients were oxycodone (71%), 8% were morphine, 7% tramadol, 4% hydrocodone, 4% codeine, 2% fentanyl, and 4% were other opioids. The number of monthly prescriptions decreased about 14% during the study period. Bivariate analyses indicated that most demographic and clinical variables were associated with receipt of any opioid prescription. After controlling for patient socio-demographic characteristics and clinical factors, the odds of receipt of any prescription were higher among patients with pain diagnoses and opioid use and mental health disorders. In addition, the odds of receipt of high average daily morphine equivalent dose (MED) prescriptions were higher for patients with pain diagnoses. Lastly, patients with substance use disorders (SUD) had an increased likelihood of detectable viral load compared to patients with no SUD, after adjusting for known covariates. Our findings show that despite opioid prescribing guidelines and monitoring systems, additional efforts are needed to prevent chronic high risk prescriptions in patients with comorbid conditions, including pain-related, mental health and substance use disorders. Evidence about the risk for chronic high risk use based on prescribing patterns could better inform pain management and opioid prescribing practices for patients receiving HIV care.

## Introduction

Despite large-scale investments at the national, state and local levels to address the opioid epidemic in the U.S., including efforts to promote judicious opioid prescribing, persons with HIV (PWH) remain at risk for adverse sequelae of prescription opioid use, including chronic opioid use, dependence, and overdosage. Studies over the last decade consistently show that PWH have a high prevalence of chronic pain at all stages of HIV and have a high prevalence of undertreatment of pain (Parker et al., 2014; Kowalski et al., 2015). Chronic pain in PWH includes the classically described syndromes of HIV associated neurologic diseases (HAND) and avascular necrosis, and also a high burden of regional and diffuse musculoskeletal pain (Robinson-Papp & Simpson, 2009; Mazzotta et al., 2011; Miaskowski et al., 2011; Merlin et al., 2016). While guidelines emphasize the primary role of non-pharmacologic and non-opioid pharmacologic interventions to promote safe and effective chronic pain management, PWH are more likely to have received opioid prescriptions, at higher doses, and for longer periods compared to the general population (Edelman et al., 2013; Canan et al., 2018; Merlin et al., 2018; Canan et al., 2019; Lemons et al., 2019). Among patients receiving HIV care, between 17 to 53% received opioid prescriptions, (Silverberg et al., 2012; Edelman et al., 2013; Koeppe et al., 2013; Jeevanjee et al., 2014; Merlin et al., 2016; Canan et al., 2018; Flores et al., 2018; Canan et al., 2019; Edelman et al., 2020), and estimates indicate that between 2-65% report misusing them (Newville et al., 2015; Lemons et al., 2019) and between 8-17% have chronic opioid prescriptions (Merlin et al., 2016). Medicaid claims data from 2001-2009 showed that the odds of chronic opioid use was 3 times higher among PWH compared to those without HIV(Canan et al., 2019). Additionally, estimates of the prevalence of opioid use disorders (OUD) among PWH show higher rates compared to people without HIV (Edelman et al., 2013; Hartzler et al., 2017). Jurisdictions and health care providers face challenges in ensuring effective chronic pain management while preventing and addressing opioid misuse, OUD and opioid related morbidity and among PWH.

PWH often present with concomitant health conditions that cause chronic pain and consequently require pain management. Evidence demonstrates that an estimated 25–80% of PWH report health conditions and disorders associated with chronic pain (Tsao et al., 2010; Dowell et al., 2016; Dowell et al., 2016; Bruce et al., 2017). These conditions may require prescription opioid use to improve overall function and well-being, if first line non-pharmacologic and non-opioid pharmacologic treatments have been unsuccessful in relieving pain and restoring function (Robinson-Papp et al., 2010; Miaskowski et al., 2011; Parker et al., 2014; Kowalski et al., 2015; Merlin et al., 2016; Merlin et al., 2018).

Concurrently, PWH have a high prevalence of multilevel risks and exposures, which may increase the likelihood that prescribed opioids may be misused or may otherwise complicate or compromise HIV treatment outcomes, such as engagement in care, adherence to antiretroviral therapy (ART), and maintenance of viral load (VL) suppression (Robinson-Papp et al., 2012). The prevalence of mental health and substance use disorders among PWH exceed those in the general population (Petry, 1999; Turner et al., 2001; Chander et al., 2006; Samet et al., 2007; Sohler et al., 2007; Tsao et al., 2007; Pence et al., 2008; Altice et al., 2010; Azar et al., 2010; Hahn & Samet, 2010; Justice et al., 2010; Tsao et al., 2011; Tsao et al., 2012; Merlin et al., 2016). Approximately half of PWH have a history of mental health or substance use disorders. Data show that 5–33% drink alcohol at hazardous levels, (Beltrami et al., 2000; Cook et al., 2001; Galvan et al., 2002; Conigliaro et al., 2003; Chander et al., 2006; Braithwaite et al., 2007; Chander et al., 2008; Bertholet et al., 2010; Green et al., 2010; Justice et al., 2010; Marshall et al., 2015; Crane et al., 2017; Bensley et al., 2018), between 22-40% report use of illicit drugs, (Bing et al., 2001; Tucker et al., 2003; Chander et al., 2008; Korthuis et al., 2012), and 8–48% meet criteria for substance disorders(Dew et al., 1997; Cook et al., 2001; Samet et al., 2007; Tsao et al., 2007; Green et al., 2010; Proeschold-Bell et al., 2010; Robinson-Papp et al., 2012; Tsao et al., 2012; Marshall et al., 2015; Newville et al., 2015; Hartzler et al., 2017; Williams et al., 2017; Williams et al., 2018). In a study of opioid prescriptions using a random sample of records of commercially insured patients in the U.S., Shah and colleagues found that transitions from initiation of prescription opioid pain management to chronic opioid use occur very quickly (Shah et al., 2017). Examination of the first month of prescriptions showed that the risk for chronic use increases within three days of initiating prescription opioid use, and the likelihood of use beyond a year doubles just after seven days of use.

However, gaps in knowledge persist about correlates of chronic high risk opioid prescriptions among PWH, (Merlin et al., 2016; Canan et al., 2018), and data of its impact on HIV outcomes are limited (Cunningham, 2018; Flores et al., 2018). Additionally, the limited studies on the impact of prescription opioid use on adverse HIV outcomes have shown conflicting results (Cunningham, 2018). Studies have found either no effect on VL suppression with prescribed opioids (Önen et al., 2012; Koeppe et al., 2013; Newville et al., 2015; Merlin et al., 2016; Canan et al., 2018; Schranz et al., 2019) or a protective effect on virologic failure (VL > 1000 copies/mL) with long-term (at least 90 consecutive days) chronic prescriptions (Merlin et al., 2018). However, in a large retrospective study that examined the association between opioid prescriptions and VL using medical records at a large healthcare system, virologic failure was more likely among patients with an opioid prescription, even after accounting for known predictors of high VL (Flores et al., 2018). Additionally, adverse outcomes (e.g., non-adherence to ART, higher VL) were found when comparisons involved repeat prescriptions or misuse of opioids(Robinson-Papp et al., 2012; Önen et al., 2012; Jeevanjee et al., 2014; Lemons et al., 2019). Mechanisms of how use of opioid analgesics impacts HIV outcomes are poorly understood (Cunningham, 2018) with assumptions about patient motivation to maintain a prescription as a potential driver for engagement and retention in HIV care and ART adherence, on one hand, and problematic opioid use as the premise for poor engagement in care and adherence, on the other hand, which is consistent with empirical evidence on substance use disorders (SUD) and adverse HIV outcomes more generally. Examination of trends in prescribing patterns and identification of factors that impact the course of prescribed opioid use among PWH are crucial, particularly given their potential to identify both trajectories that move from short term low risk use to chronic high risk use and how these, in turn, may impact HIV outcomes (Flores et al., 2018).

This study sought to characterize opioid prescription patterns and identify risk factors for chronic high risk opioid prescriptions and HIV outcomes among PWH in primary care. The study involved electronic medical records (EMR) from a large health system with a comprehensive HIV treatment center in NYC during a period following the dissemination of a set of opioid prescribing guidelines designed to curtail the epidemic. In March 2016, the CDC updated the 2014 national recommendations on opioid prescriptions for primary care clinicians treating adult patients with non-cancer chronic pain specifying the importance of risk assessments, prescription initiation or continuation, appropriate drug and dosing, and ongoing assessments for and linkage to OUD and SUD treatment (Dowell et al., 2016; Dowell et al., 2016). Additionally, laws in NY State were updated in 2016 to limit prescriptions to seven days for acute pain, adding to the 2013 mandate for the state’s prescription monitoring program (I-Stop) for physicians to review a patient’s opioid prescription history and set of recommendations for patients discharged from emergency departments (Public Health Article 33, 2016). Thus, this study was uniquely positioned to examine trends in opioid prescribing practices to assess short-term impact of public health policies to curb the opioid epidemic.

## Methods

### Data Source

EMR data from patients receiving HIV care at the Mount Sinai Institute for Advanced Medicine (IAM) in New York City were extracted for retrospective analysis. The IAM is comprised of five ambulatory care centers which provide comprehensive care to persons with HIV who are predominately uninsured or receive federal or state assistance (e.g. Medicaid, Medicare, and Ryan White). The IAM provides primary care and specialty care in cardiology, dermatology, nephrology, neurology and psychiatry, as well as support services for mental health and social services, case management, and coordinated clinical care. EMR documented clinical encounters occurring between July 1, 2016–December 31, 2017 for patients who met the following study inclusion criteria we included for analysis: 1) age ≥18 years, 2) confirmed HIV diagnosis, and 3) at least one primary care visit during the study period.

EMR were collected using Microsoft Access to query Epic Clarity, a reporting database that interfaces with the Epic EMR system. Data tables containing patient- and encounter-level records were managed in Access. De-identified datasets were imported into SPSS (version 24) for data cleaning and analysis. Manual chart reviews of subsets of cases were conducted to verify records as needed. The Institutional Review Board at Mount Sinai approved procedures for this study.

### Variables Extracted

Patient socio-demographic data extracted included age, gender, ethnicity and race. Age was not normally distributed and was skewed with 74% of patients being 40 years of age or older and thus, we categorized the variable into 4 age groups (18–29, 30–39, 40–49, and 50+) and also dichotomized (<40 and ≥40). Gender was collapsed into 3 groups from the 4 gender identity groups attained from the EMR: 1) cisgender and transgender male (*n* = 6,846); 2) cisgender female (*n* = 1,970); and 3) transgender female (*n* = 66). Due the small number of transgender males (*n* = 3), records were combined with those of cisgender males (*n* = 6,843) for a total of 6,846. Ethnicity and race were combined into one variable and collapsed as non-Hispanic African-American or Black, Hispanic, non-Hispanic White, Asian or Pacific Islander, and mixed or other.

The variable “years since HIV diagnosis” was not normally distributed and had a platykurtic distribution with negative kurtosis values. Thus, the variable was categorized into 3 groups based on the number of years with an HIV diagnosis (<5 years, between 5 and <10 years, and ≥10 years). Chart reviews were conducted to verify diagnosis date for most patients; however, diagnosis dates could not be determined for 2,430 patients. Diagnosis codes based on International Classification of Diseases-9/10-Clinical Modification (ICD-9/10-CM) codes were utilized to identify deceased patients and patients with non-opioid SUD, OUD, mental health disorders (adjustment, anxiety, bipolar, depression, eating, gender identity, neurocognitive, neurodevelopmental, obsessive compulsive, personality, psychotic, sexual, sleep, trauma), and pain disorders. Data on recent substance use (in the 6 months prior to clinic visit) were extracted from substance use screening, completed at the time of an encounter. Providers or clinical team members asked patients at the time of a primary care visit about the use of different illicit substances and misuse of prescriptions and alcohol. Viral load and CD4 count were recorded using the first laboratory result available during the study period, and the variables were categorized into virally suppressed (<50 copies/mL) vs. unsuppressed (≥50 copies/mL) and CD4 counts were dichotomized as <200 or ≥200.

A total of 18,296 records of opioid analgesic prescriptions, excluding any methadone and buprenorphine formulations, prescribed to eligible patients were extracted from EMR. Given that we could not determine from the extracted EMR whether methadone and buprenorphine were prescribed to treat pain vs. to treat opioid use disorders, as they are commonly prescribed, we excluded records of patients receiving methadone or buprenorphine for the purposes of this study. Prescription records included brand and generic name, date ordered, start and end date of prescription course, dosage, and quantity to be dispensed (e.g., number of tablets). Some prescriptions documented in the EMR had a ‘discontinuation indicator, with categories including discontinued by another clinician or patient; patient refusal, non-compliance or transfer; changed due to drug interactions or side effects; formulary or dose change; and indication that therapy was completed; or prescription entry errors. We then validated opioid prescriptions through a multi-step chart review process. For a record to be determined to be valid, either of the following criteria had to be met: an electronically confirmed receipt by the pharmacy, or a prescription for opioids was refilled during the study period. A subsample of 50 records was randomly selected for each discontinuation category and chart reviewed to determine whether each of those prescriptions was valid. If all of the records in the subsample for a particular discontinuation category were found to be valid, then all of the prescriptions for that category were considered valid and included in analysis (this was the case for the categories discontinued by another clinician, alternate therapy, side effects, duplicate medication, and entry error). If not all records in the subset of any given discontinuation category were considered valid, then the full dataset of records for those specific categories were chart reviewed to determine validity for each prescription record (this was the case for the categories patient discontinued, dose adjustment, and “other”). A total of 7,548 (41.3%) of all opioid prescriptions were deemed invalid (for the reasons delineated above) and were excluded. Additionally, 2,004 prescription records were excluded for patients with a cancer diagnosis. Thus, a total of 8,744 prescriptions were included for data analysis.

To account for differences in opioid drug type and dose, we calculated each patient’s average daily morphine equivalent dose (MED) based on CDC’s standardized measure that considers morphine conversion factor for the opioid drug type, dose, number dispensed, and days supplied (Dowell et al., 2016; HHS Office of Inspector General, 2020). Of the 8,744 opioid prescriptions included in analysis, 1,508 (17.3%) prescription records had to be excluded in calculating the MED variable because start and end dates for those prescriptions had incomplete or erroneous information. Additionally, MED was not computed for 186 (2.1%) transdermal prescriptions. For 7,050 records with valid prescription dates, we aggregated the total number of days supplied for each patient. Long term opioid prescriptions were defined as those extending >365 days.

### Outcomes

The three outcomes were receipt of any opioid prescription, receipt of chronic high MED, and unsuppressed VL. We defined receipt of any opioid prescription as a record of an opioid analgesic prescription regardless of days supplied or dosage. Chronic high MED prescription receipt was defined as having received a prescription with daily dosage of >120 MED for ≥30 consecutive days (Dowell et al., 2016; Canan et al., 2018; Merlin et al., 2018; HHS Office of Inspector General, 2020). Unsuppressed VL was defined as having a viral load ≥50 copies/mL (Thompson et al., 2020).

### Data Analysis

Descriptive statistics were calculated with percentages for categorical variables and means and standard deviations (SDs) for continuous variables. Opioid prescriptions were aggregated by month and trends in prescribing (number of prescriptions per month, median MED per month, and percentage of prescriptions per month) were summarized by subgroup, in terms of age group, gender, race/ethnicity, and pain diagnosis, differentiating all subgroups by low (≤120) and high (>120) MED. Chi-square tests for bivariate analyses examined differences in characteristics of patients who had or had not received ≥1 opioid prescription. Bivariate associations between each outcome and patient-level factors were examined in separate unadjusted logistic regressions. To identify patient-level factors associated with each outcome, separate stepwise multivariate linear regression models were conducted incorporating age, gender, race/ethnicity, years since HIV diagnosis as a categorical variable, pain diagnosis, SUD, OUD, and mental health disorders. The model predicting high VL also included the effect of MED to compare high MED prescriptions to no prescriptions and low MED. Statistical analyses were performed in SPSS version 24.

## Results

### Socio-Demographic and Clinical Characteristics

Table 1 presents the demographic and clinical characteristics of the 8,882 patients who had at least one HIV primary care visit from July 1, 2016 - December 31, 2017. Patients were predominantly male and ethnically and racially diverse. Half of the sample was 50 years of age or older with 39% having been diagnosed with HIV for 10 or more years. About 80% were virally suppressed (<50 copies/mL) and 91% had a CD4 count ≥200 based on the first available laboratory result during the study period. Additionally, 28% had documentation of a pain diagnosis, and nearly 39% had a mental health disorder, including depressive (21%), anxiety (13%), sleep (6%), bipolar (4%), and adjustment disorders (3%). EMR documentation of recent substance use during primary care visits was made for approximately 30% of patients (2694/8882); 16% (454/2694) of those screened reported alcohol or drug use in the 6 months prior to a visit, representing 5% (454/8882) of all patients.

**TABLE 1:** Socio-Demographic and Clinical Characteristics of People in HIV Care who Received ≥1 Opioid Prescription, July 2016-December 2017 (*n* = 8,882).

	Any opioid prescription						
	Total		Yes		No		
	(*n* = 8,882)		(*n* = 1,040, 11.7%)		(*n* = 7,842, 88.3%)		
	N	(%)	n	(%)	n	(%)	*p*
Age, years (M, SD, min-max)	(48.02,12.68,18–92)		(53.61,10.70,19–85)		(47.27,12.74,18–92)		
18–29	848	9.5	36	4.2	812	95.8	***
30–39	1674	18.8	81	4.8	1593	95.2	
40–49	1897	21.4	186	9.8	1711	90.2	
50+	4463	50.2	737	16.5	3726	83.5	
Gender							
Male	6846	77.1	654	9.6	6192	90.4	***
Cisgender female	1970	22.2	368	18.7	1602	81.3	
Transgender female	66	0.7	18	27.3	48	72.7	
Ethnicity/Race							
Non-hispanic african-american	2988	33.6	377	12.6	2611	87.4	***
Hispanic	2163	24.4	326	15.1	1837	84.9	
Asian or pacific Islander	151	1.7	5	3.3	146	96.7	
Other/Multiple	1680	18.9	157	9.3	1523	90.7	
Non-hispanic white	1900	21.4	175	9.2	1725	90.8	
Years since HIV diagnosis (M, SD, min-max)	(12.57,9.15,0–41.50)		(16.40,9.08.02–36.5)		(12.06,9.04,0–41.5)		
<5 years	1836	20.7	108	5.9	1728	94.1	***
5 < 10 years	1388	15.6	148	10.7	1240	89.3	
≥10 years	3449	38.8	523	15.2	2926	84.8	
Missing	2209	24.9					
Death during study period							
Yes	46	0.5	15	32.6	31	67.4	***
No	8636	99.5	1025	11.6	7811	88.4	
HIV viral suppression							
Suppressed (<50 copies/mL)	7048	79.4	815	11.6	6233	88.4	ns
Unsuppressed (≥50 copies/mL)	1698	19.1	218	12.8	1480	87.2	
Missing	136	1.5					
CD4							
<200 cells/mL	625	7.0	106	17.0	519	83.0	***
≥200 cells/mL	8081	91.0	925	11.4	7156	88.6	
Missing	176	2.0					
Pain diagnosis							
Yes	2522	28.4	610	24.2	1912	75.8	***
No	6198	69.8	418	6.7	5780	93.3	
Substance use disorder (excluding opioid use disorder)							
Yes	1428	16.1	213	14.9	1215	85.1	***
No	7454	83.9	827	11.1	6627	88.9	
Opioid use disorder							
Yes	367	4.1	99	27.0	268	73.0	***
No	8515	95.9	941	11.1	7574	88.9	
Substance use in past 6 months							
Yes	454	5.1	63	13.9	391	86.1	ns
No	2240	25.2	306	13.7	1934	86.3	
Missing	6188	69.7					
Mental health disorder							
Yes	3426	38.6	526	15.4	2900	84.6	***
No	5456	61.4	514	9.4	4942	90.6	

a Includes 6,843 cisgender and 3 transgender males; *p < .05; **p < .01; ***p < .001.

M = mean; SD = standard deviation; min-max = minimum and maximum values; ns = nonsignificant.

Overall, 1631 (18%) patients had documentation of ≥1 SUD; of those, 23% had OUD, 31% alcohol, 31% cocaine, 23% cannabis, 18% amphetamine use disorders, and 19% other or unspecified substances (results not shown in tables). A greater percentage of men had documentation of an alcohol or an amphetamine use disorder compared to cisgender and transgender women (6 vs. 4% and 3% for alcohol; 4 vs. 0.3% and 1% for amphetamine, respectively; all *p* < 0.01), while a greater percentage of cisgender and transgender women had documentation of an OUD excluding heroin compared to men (5 and 5% vs. 3% for opioid, respectively; all *p* < 0.05, results not shown in tables).

### Opioid Prescription Trends

**FIGURE 1:**
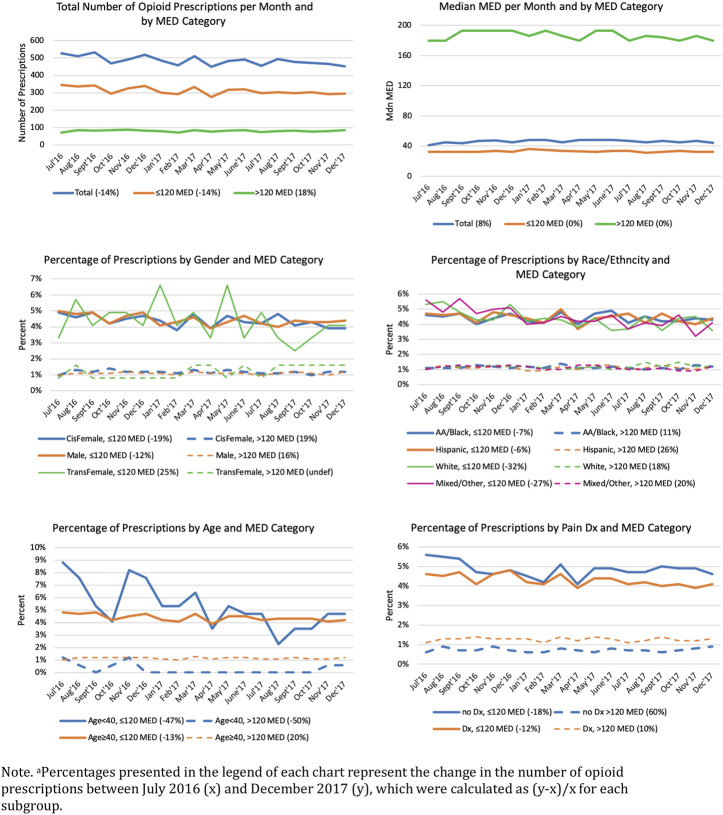
Monthly Opioid Prescriptions and Average Daily Morphine Equivalent Dose (MED) Trajectories by Patient Demographic and Clinical Characteristics^a^. Note. ^a^Percentages presented in the legend of each chart represent the change in the number of opioid prescriptions between July 2016 (x) and December 2017 (y), which were calculated as (y–x)/x for each subgroup.

During the 18-month study period, 8,744 opioid prescriptions (98% oral and 2% transdermal opioid preparations) were provided to 1,040 (12%) patients (results not shown in tables). The majority (71%) of prescriptions were oxycodone, 8% were morphine, 7% tramadol, 4% hydrocodone, 4% codeine, 2% fentanyl, and 4% were other opioids. The median daily dosage was 46.55 MED (M = 75.55, SD = 89.80); 5,606 (80%) prescriptions were low MDE and 1,444 (20%) were high MDE. Figure 1 depicts trends in monthly prescriptions by age group, gender, race/ethnicity, and pain diagnosis by low and high MED subgroups. Over the study period, the number of monthly prescriptions decreased by 14.1%, from 526 prescriptions in the first month (July, 2016) to 452 prescriptions in last month (December, 2017). However, while low MED (≤120) decreased by 14.2% from 345 to 296 prescriptions, high MED increased by 18.3% from 71 to 84 prescriptions. Median MED decreased by 7% overall from 48.21 to 44.72; however, the decrease occurred in the low MED subgroup; the median MED among the high MED subgroup remained stable during the study period. Percentages of monthly prescriptions by age, gender and race/ethnicity, and pain diagnosis showed similar trends among subgroups with the exception of inflections in prescriptions among younger (<40 years) patients and transgender women.

### Univariate Analyses

Among all patients, 1040 (12%) patients had at least one opioid prescription; 8% of patients received prescriptions for oxycodone, 2% for tramadol, and 2% for other opioids including codeine, fentanyl, hydrocodone, hydromorphone, morphine, oxymorphone, tapentadol, and tramadol (results not shown in tables). Among those with a prescription, 36% of patients received just one opioid prescription, 20% had 2-3 prescriptions and 44% had ≥4 prescriptions during the 18-month study period. Bivariate analyses indicated that most demographic and clinical variables, with the exception of VL suppression, were associated with receipt of any opioid prescriptions (Table 1). Older age and identifying as *cis*-gender female, transgender female, Black/African American or Hispanic were associated with receipt of any prescriptions. Similarly, having been diagnosed with HIV for 10 or more years, a CD4 count <200, and documentation of having died during the study period were associated with having received ≥1 prescription. Furthermore, having documentation of a mental health disorder, an OUD, and a SUD including alcohol or drugs other than opioids were each associated with having received ≥1 prescription. Among the 2694 patients who were asked about recent substance use during their primary care visit, a similar percentage of patients (∼14%) with or without recent substance use received prescriptions. No significant associations were found between having received prescriptions and use of specific substances, except that a lower percentage of patients who reported using crystal methamphetamine received prescriptions compared to patients with no crystal methamphetamine use (5 vs. 14%, *p* = 0.03).

Analyses of daily dosage of >120 MED and long-term opioid prescription receipt (>1 year) showed several statistically significant trends among the patients with available prescription data. Long-term opioid prescriptions were found among 311 (40%) of 776 patients. Nearly 43% of patients 40 years of age or older had prescriptions for a year or longer compared to 5% of younger patients (*p* < 0.001). A larger percentage of patients with long-term prescriptions had diagnosed HIV longer than 5 years (47% 10 or more years, 28% between 5 and 10 years and 16% less than 5 years; *p* < 0.001) and had a pain diagnosis (49 vs. 26% no pain diagnosis, *p* < 0.001). Additionally, of the 772 patients with available MED data, 109 (14%) patients received a high daily dose and 663 (86%) patients had prescriptions with a daily dosage ≤120 MED Receipt of chronic high MED opioid prescriptions was associated with older age and pain diagnosis. More patients ≥40 years of age received a high-dose MED than did those under 40 (15 vs. 4%, *p* < 0.05). Additionally, a larger percentage of patients with a pain diagnosis received a high MED than patients without a pain diagnosis (18 vs. 8%, *p* < 0.001). Neither viral suppression nor CD4 count was associated with high MED or long-term opioid prescriptions.

### Multivariate Analyses

Separate multivariate analyses examined which factors were independently associated with either receipt of any opioid prescription, receipt of high MED, or VL nonsuppression (Table 2). Logistic regression controlling for age group, gender, race/ethnicity, and years since HIV diagnosis showed that the presence of pain diagnosis, OUD and mental health disorders were independently associated with receipt of any opioid prescription. Among patients with prescriptions, adjusted logistic regression examining MED indicated increased odds of a chronic high-dose prescription among patients with pain diagnoses compared to patients without those diagnoses. The last model examined viral suppression and found that SUD increased the likelihood of detectable VL after accounting for demographic and clinical factors. Having a chronic high MED was not found to be independently associated with having a detectable VL when compared to those who were not prescribed opioids and those with low MED opioid prescriptions.

**TABLE 2:** Unadjusted and Adjusted Logistic Regression Analyses of Factors Associated with Receipt of Opioid Prescription, High average daily morphine equivalent dose (MED) Prescription, and with Unsuppressed HIV Viral Load.

		Any opioid prescription		High MED opioid prescription		Unsuppressed HIV viral load	
		Unadjusted (*n* = 8,882)	Adjusted (*n* = 6,579)^a^	Unadjusted (*n* = 772)	Adjusted (*n* = 570)^b^	Unadjusted (*n* = 8,746)	Adjusted (*n* = 6,302)^a^
		OR (95% CI)	AOR (95% CI)	OR (95% CI)	AOR (95% CI)	OR	AOR (95% CI)
Age <40 years		0.29 (0.24–0.35)***	0.49 (0.38–0.64)***	0.21 (0.05–0.86)*	0.16 (0.02–1.31)	1.15 (1.02–1.28)*	1.14 (0.97–1.34)
Gender							
	Male^c^	0.46 (0.40–0.53)***	0.54 (0.46–0.64)***	0.90 (0.59–1.38)	1.25 (0.72–2.14)	0.76 (0.67–0.86)***	0.88 (0.76–1.03)
	Transgender female	1.66 (0.95–2.88)	2.05 (1.01–4.16)*	1.73 (0.46–6.57)	4.45 (0.96–20.59)	0.91 (0.50–1.65)	0.93 (0.45–1.94)
	Cisgender female	Ref	Ref	Ref	Ref	Ref	Ref
Ethnicity/Race							
	African-american	1.42 (1.18–1.72)***	1.12 (0.89–1.41)	1.27 (0.69–2.35)	1.69 (0.75–3.83)	2.25 (1.92–2.63)***	2.14 (1.78–2.58)***
	Hispanic	1.75 (1.44–2.13)***	1.19 (0.94–1.51)	1.00 (0.53–1.90)	1.31 (0.56–3.06)	1.30 (1.09–1.55)**	1.24 (1.00–1.53)*
	Other/Multiple	0.96 (0.77–1.20)	0.85 (0.65–1.11)	1.08 (0.51–2.27)	1.51 (0.58–3.96)	1.65 (1.38–1.97)***	1.62 (1.32–2.00)***
	White	Ref	Ref	Ref	Ref	Ref	Ref
Years since HIV diagnosis							
	<5 years	0.35 (0.28–0.43)***	0.69 (0.54–0.87)**	0.95 (0.46–1.95)	1.49 (0.69–3.24)	1.15 (1.00–1.33)*	1.14 (0.96–1.34)
	5 < 10 years	0.67 (0.55–0.81)***	0.80 (0.65–0.98)*	0.53 (0.24–1.14)	0.58 (0.26–1.27)	1.02 (0.87–1.19)	1.02 (0.86–1.21)
	≥10 years	Ref	Ref	Ref	Ref	Ref	
Pain diagnosis		4.41 (3.86–5.05)***	3.36 (2.85–3.95)***	2.63 (1.59–4.34)***	2.49 (1.35–4.62)**	0.95 (0.85–1.07)	0.89 (0.77–1.04)
SUD (excluding OUD)		1.41 (1.19–1.65)***	1.07 (0.87–1.31)	0.82 (0.49–1.37)	0.67 (0.34–1.30)	1.72 (1.51–1.96)***	1.79 (1.52–2.11)***
OUD		2.97 (2.34–3.78)***	2.22 (1.63–3.01)***	1.11 (0.59–2.09)	1.14 (0.53–2.48)	1.41 (1.10–1.79)**	1.31 (0.96–1.80)
Mental health disorder		1.74 (1.53–1.99)***	1.31 (1.11–1.54)**	1.05 (0.70–1.57)	1.20 (0.70–2.04)	1.06 (0.95–1.19)	0.98 (0.86–1.13)
MED							
	No opioid prescription	-	-	-	-	0.68 (0.44–1.05)	0.67 (0.39–1.15)
	Low MED	-	-	-	-	0.75 (0.47–1.20)	0.72 (0.41–2.29)
	High MED					Ref	Ref

a Excludes patients with missing data mostly due to missing HIV diagnosis date.

b Excludes patients with missing MED data and HIV diagnosis date.

c Includes 6,843 cisgender and 3 transgender males.

*p < .05; **p < .01; ***p < .001

SUD = substance use disorder; OUD = opioid use disorder; MED = average daily morphine equivalent dose; OR = odds ratio; AOR = adjusted odds ratio.

## Discussion

We characterized trends in opioid prescription patterns among patients receiving HIV care over 18 months following the publication of the 2016 CDC pain management guidelines, identified risk factors for chronic high risk opioid prescriptions, and examined the impact of prescriptions on virologic suppression. Of the 8,882 patients receiving HIV care, 12% had at least one opioid prescription during the study period, which was lower than estimates of between 17 to 53% found in prior studies (Silverberg et al., 2012; Edelman et al., 2013; Koeppe et al., 2013; Jeevanjee et al., 2014; Merlin et al., 2016; Canan et al., 2018; Flores et al., 2018; Canan et al., 2019; Edelman et al., 2020). During the study, the majority (56%) of patients with an opioid prescription received 1-3 prescriptions and 40% received long-term (>365 days) prescriptions. In adjusted analysis, the presence of pain diagnosis, OUD and mental health disorders were independently associated with an increased likelihood of having received at least one opioid prescription. Overall, the number of monthly prescriptions decreased by 14% from the first month to the last month of the 18-month period, and this decrease occurred primarily among low MED prescriptions, which represented most (80%) of the prescriptions during the study. However, a 18% increase was observed in receipt of chronic high dose prescriptions (>120 MED), representing about 20% of prescriptions during the study. Taken together, these data suggest that public health guidelines and regulations that directly address prescribing practices can have an impact in reducing the overall number of prescriptions, at least in the immediate short-term, but additional approaches may be needed to specifically address initiation of high MED and transitions from low to high dose and high risk prescriptions.

Among patients receiving any opioid prescriptions, chronic high risk opioid prescriptions were provided to 14% of those with available MED data, which is similar to Merlin et al.’s estimate of 17% of patients with chronic prescriptions, (Merlin et al., 2016), but higher than estimates in earlier studies (between 6-10%) (Silverberg et al., 2012; Edelman et al., 2013). However, using four separate indicators for high risk opioid prescriptions based on national criteria for prescription monitoring, Canan and colleagues found that 30%, a substantial larger percentage of PWH compared to our finding (14%), met criteria for high risk opioid prescriptions (Canan et al., 2018). In our study, only pain diagnoses were found to predict high MED prescriptions in adjusted logistic regression analysis. The likelihood of chronic high risk opioid prescriptions was elevated in patients with pain diagnoses compared to patients without those diagnoses. Our findings are similar to those found in the study conducted by Canan and colleagues (2018), which found that pain diagnoses were associated with high risk prescriptions but no associations between high risk prescriptions and other factors, such as mental health disorders or viral suppression (Canan et al., 2018). Importantly, in our study, having documentation of SUD, not including OUD, was predictive of viral nonsuppression, after controlling for all other variables in the model. Both receipt of an opioid prescription and receipt of a high MED prescription were not significantly associated with viral suppression. A similar percentage of patients with suppressed and nonsuppressed VL were likely to receive any opioid prescription or high MED prescriptions. In contrast to our findings, Flores et al. found that virologic failure was more likely among patients receiving any opioid prescription, after accounting for known predictors of high VL (Flores et al., 2018).

These findings have important implications for the prevention of chronic high risk prescriptions in the clinical care of PWH with comorbid conditions, including pain-related, mental health and substance use disorders. It is important to note that EMR documentation of recent substance use during a primary care visit was made for only about 30% of patients and thus, recent substance use may be underestimated. This issue has been found in prior studies; documentation was unavailable for 37% of patients who received opioid prescriptions in the study conducted by Flores and colleagues (Flores et al., 2018). While the identified gaps in substance use screening is an important finding, indicating the need for effective strategies to enhance substance use screening rates, EMR are key to facilitate the integration of services for substance use within HIV primary care (Tai et al., 2012; Ghitza et al., 2013).

One set of important interventions has been the development and dissemination of opioid prescribing guidelines and pain management strategies which emphasize that non-pharmacologic interventions, and non-opioid pharmacologic interventions should be considered first line, to reduce risk for complications of opioid use among PWH (Bruce et al., 2017; Flores et al., 2018). Qualitative studies focusing on the training needs of HIV treatment providers, such as the study conducted by Starrels and colleagues (Starrels et al., 2016) could help in the development of interventions and programs to enhance the implementation of and adherence to evidence-based practices grounded in consensus opioid prescribing and pain management guidelines. Several efforts are currently underway to improve knowledge and training about pain management, co-occurring mental health and SUD, and pharmacotherapy, as well as to facilitate screening, monitoring and judicious prescribing among HIV primary care providers (Lum et al., 2011; Bruce et al., 2017). Robinson-Papp and colleagues developed an innovative intervention, TOWard SafER Opioid Prescribing (TOWER), (Robinson-Papp et al., 2019), to support providers in applying CDC’s guidelines with their patients by providing tools in the management of pain and opioid prescribing practice. Patients use an SMS-based app daily to record pain intensity and benefits of treatment, adverse reactions and harms, and non-pharmacologic treatment and opioid use in order to provide data for a risk-benefit assessment. Patient’s responses, along with EMR and records from prescription monitoring programs are summarized in an app that providers use to support patients managing their pain. Focusing on chronic opioid use and HIV treatment, Lira and colleagues developed the Targeting Effective Analgesia in Clinics for HIV (TEACH) intervention to support providers by providing access to a specialized nurse care manager, an addiction specialist, and prescriber education and training (Lira et al., 2019). Other educational efforts are already proving to have an effect in improving knowledge among providers about prescribing guidelines in NYC regarding acute pain, chronic noncancer pain, and the dangers of high-dose opioid prescriptions (Kattan et al., 2016).

### Limitations

This study has important limitations which need to be considered. First, although this study was based on large retrospective dataset over an 18-month period, we aggregated individual prescriptions for each patient, and therefore, we did not conduct analyses to make any causal inferences. While we were able to determine associations between individual demographic and clinical indicators and opioid prescriptions, observational studies cannot demonstrate causation and therefore, associations should be interpreted with caution. Second, there are inherent limitations to EMR data because they are dependent on documentation of the clinical encounter, prescriptions made, and whether patients are asked about or report symptoms or problems beyond their immediate HIV care needs. As noted above documentation of recent substance use during a primary care visit was made for only about 30% of patients and thus, recent substance use may be underestimated. Additionally, reliance on ICD diagnostic codes to define the various disorders has limitations as some conditions may be undiagnosed or otherwise not recorded, including pain diagnoses. Furthermore, because we could not readily determine from the extracted EMR whether methadone and buprenorphine were prescribed to treat pain vs. to treat opioid use disorders, we excluded records of patients receiving methadone or buprenorphine. Third, we did not interview or conduct any assessments with patients. Although bias related to recall and social desirability is introduced with self-reports, we could not assess whether the opioid prescriptions documented in EMR were dispensed by the pharmacy, if patients complied with doctor’s orders and took them as prescribed, or whether additional opioid prescriptions were dispensed by providers outside the healthcare system in this study. The results of this study may not generalizable to PWH not retained in HIV care, and given that a large percentage of patients in our sample were virally suppressed, the results may also not be generalizable to all PWH in HIV care. We were also limited in the records that were attained from EMR system and could not follow up directly with patients to fully obtain the diversity of the sample of patients. This limitation is most apparent in our approach of collapsing the gender identity variable. Additionally, EMR included in this study were not from a nationally representative sample of patients; however, the sample was very diverse in terms of gender and race/ethnicity.

## Conclusion

Our findings show that receipt of opioid prescriptions, including high MED, and therefore potentially high risk, opioid prescriptions, including receipt of prescriptions for long durations, are common among PLW who are in care. Despite large-scale efforts to improve prescribing guidelines and implement monitoring systems, additional efforts are needed for PWH to support patients and clinicians in reducing chronic high risk use of prescription opioids, particularly for patients with co-occurring pain-related, mental health and substance use disorders. More recently, the NIH has advanced an initiative called Helping to End Addiction Long-term (HEAL) for addiction and pain research to treatments for problematic opioid use and pain (see www.heal.nih.gov) (Collins et al., 2018) Examination of trends in prescribing patterns and identification of factors that impact the course of opioid use among PWH are crucial, particularly given their potential to identify both, trajectories that move beyond use and misuse to OUD, and prevention and treatment targets within healthcare systems. Future research is needed to better characterize transitions to chronic high risk use to inform pain management and opioid prescribing practices for patients receiving HIV care to simultaneously improve pain management, to optimize HIV outcomes including viral suppression, and to reduce the risks of prescription opioid use.

## Data Availability

The raw data supporting the conclusions of this article will be made available by the authors, without undue reservation.
